# Supplementary material for: Treatment Monitoring of a Patient with Synchronous Metastatic Angiosarcoma and Breast Cancer Using ctDNA
Source: Int J Mol Sci. 2024 Apr 4;25(7):4023. doi: 10.3390/ijms25074023 (PMC11012383; doi:10.3390/ijms25074023)
Supplement: Supplementary file 1 [file ijms-25-04023-s001.zip › Supplementary figures.pdf]

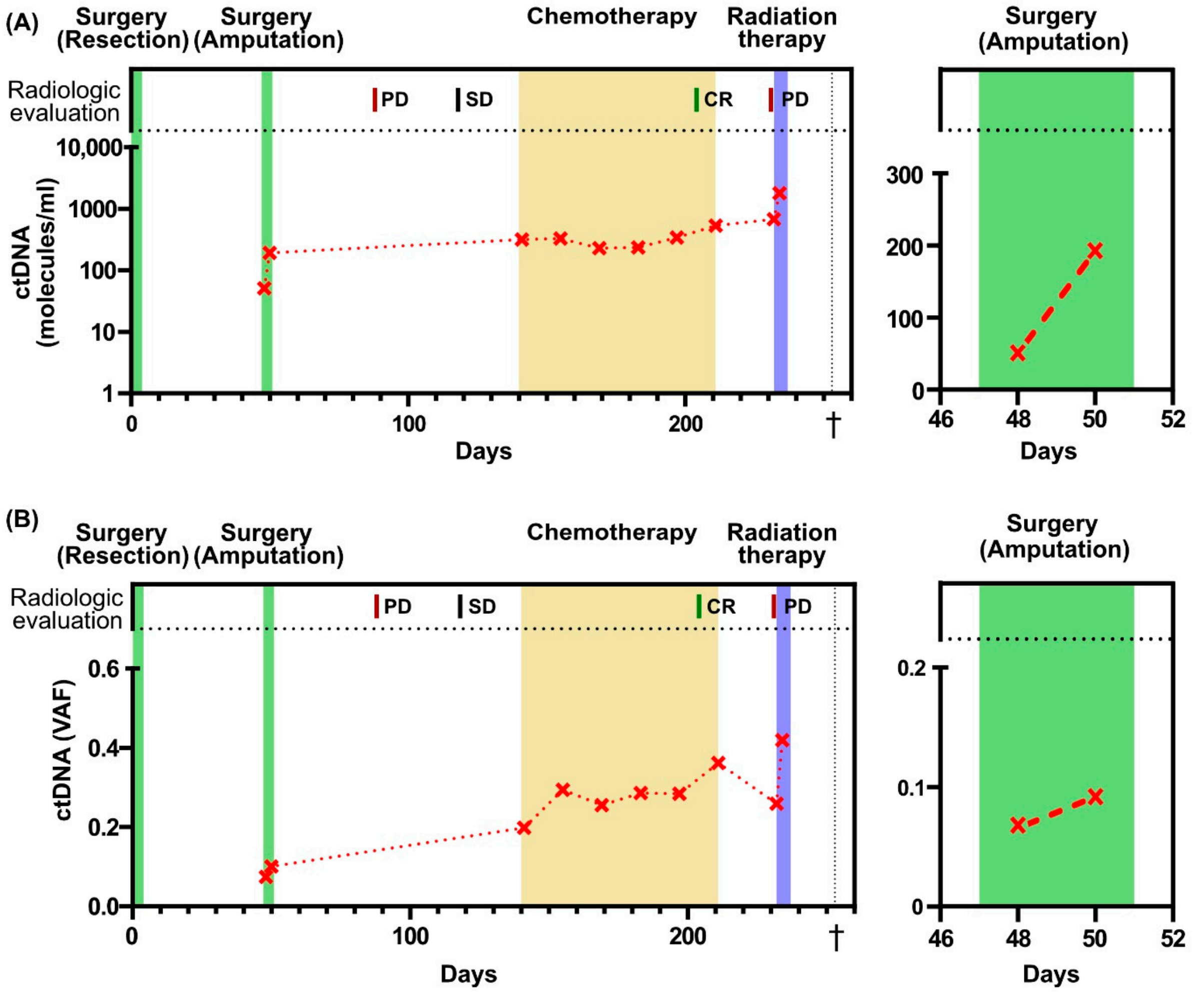

Supplementary Figure S1 – Average levels of blood-based markers over time. A) The average levels of circulating tumor-DNA (ctDNA) expressed as number of molecules per ml plasma. Treatments and radiological evaluations displayed at the top. On the right, a magnified view of the second surgery, spanning from day 46 to day 52. B) The average levels of ctDNA expressed as variant allele frequency (VAF). On the right, a magnified view of the second surgery, spanning from day 46 to day 52. PD, progressive disease; SD, Stable disease; CR, complete remission; PREOP2, blood sample collected the day before the second surgery; POSTOP2, blood sample collected the day after the second surgery; P1-P6, blood samples collected before each cycle of palliative chemotherapy; RT, blood sample collected the day before initiation of radiation therapy; RT-1, blood sample collected the day after initiation of radiation therapy; RX, resection; PX, tumor biopsy. The dagger (†) indicates when the patient deceased.
